# Leveraging Representation Learning for the Construction and Application of a Knowledge Graph for Traditional Chinese Medicine: Framework Development Study

**DOI:** 10.2196/38414

**Published:** 2022-09-02

**Authors:** Heng Weng, Jielong Chen, Aihua Ou, Yingrong Lao

**Affiliations:** 1 State Key Laboratory of Dampness Syndrome of Chinese Medicine Second Affiliated Hospital of Guangzhou University of Chinese Medicine Guangzhou China; 2 School of Information Science Guangdong University of Finance & Economics Guangzhou China

**Keywords:** knowledge graph, knowledge embedding, traditional Chinese medicine, knowledge discovery, medicine, clinical, framework

## Abstract

**Background:**

Knowledge discovery from treatment data records from Chinese physicians is a dramatic challenge in the application of artificial intelligence (AI) models to the research of traditional Chinese medicine (TCM).

**Objective:**

This paper aims to construct a TCM knowledge graph (KG) from Chinese physicians and apply it to the decision-making related to diagnosis and treatment in TCM.

**Methods:**

A new framework leveraging a representation learning method for TCM KG construction and application was designed. A transformer-based Contextualized Knowledge Graph Embedding (CoKE) model was applied to KG representation learning and knowledge distillation. Automatic identification and expansion of multihop relations were integrated with the CoKE model as a pipeline. Based on the framework, a TCM KG containing 59,882 entities (eg, diseases, symptoms, examinations, drugs), 17 relations, and 604,700 triples was constructed. The framework was validated through a link predication task.

**Results:**

Experiments showed that the framework outperforms a set of baseline models in the link prediction task using the standard metrics mean reciprocal rank (MRR) and Hits@N. The knowledge graph embedding (KGE) multitagged TCM discriminative diagnosis metrics also indicated the improvement of our framework compared with the baseline models.

**Conclusions:**

Experiments showed that the clinical KG representation learning and application framework is effective for knowledge discovery and decision-making assistance in diagnosis and treatment. Our framework shows superiority of application prospects in tasks such as KG-fused multimodal information diagnosis, KGE-based text classification, and knowledge inference–based medical question answering.

## Introduction

### Background

Having a long history of 5000 years, traditional Chinese medicine (TCM) is featured as the scientific thinking of holistic view and syndrome differentiation, as well as the long-time practice of technical methods of personalized treatment. TCM has the advantages of precise clinical efficacy, relatively safe medication, flexible treatment, and relatively low cost [1]. However, a large amount of empirical knowledge exists with Chinese physicians, which is difficult to be applied directly in assisting clinical decision-making systems. At the same time, the dismantling of medical guidelines alone cannot cope with all situations, and existing clinical assisted decision-making systems cannot explain the ins and outs of diagnostic decisions as senior experts do.

The combination of knowledge graphs (KGs) and artificial intelligence (AI) models has the bilateral advantages of “black box” and “logic.” Using knowledge graph embedding (KGE) techniques, KGE models may partially simulate the cognitive process of the human brain by representing massive entities, relations, and attributes. By combining with the causal events extracted from the text of event descriptions by causality extraction techniques, event information can be presented in structured form. KGs and machine learning models are expected to be integrated to assist machine understanding and concept interpretation, allowing the decision-making process of machines to be interpretable. However, how to construct a TCM KG and apply it with KGE models is still a challengeable problem.

To that end, this paper proposes a new framework leveraging a representation learning method for TCM KG construction and application. TCM knowledge is extracted from Chinese physicians based on 1 of our previous works [2] by using an automatic procedure of information extraction concept normalization, *entity alignment*. The framework collects multimodal information about Chinese medicines to support the automatic construction of personalized KGs according to clinical disease treatments by Chinese physicians. Our framework has application potential in text classification, KG-based question answering, and recommendations of practitioners and specialties.

The main contributions of this paper are threefold: (1) A new framework for the construction and application of TCM KG by leveraging representation learning is proposed, (2) a transformer-based Contextualized Knowledge Graph Embedding (CoKE) model is applied to KG representation learning and knowledge distillation by integrating multihop relations, and (3) a TCM KG containing 59,882 entities, 17 relations, and 604,700 triples is constructed.

### Related Work

#### Medical Knowledge Graph

The concept of KG was proposed by Google in 2012. Research applications evolved by previously improving the capabilities of search engines and enhancing the search quality and experience of users related to finance, healthcare, geography, e-commerce, and medical care. There exist many KGs, including on Google Knowledge Graph [3], DBpedia [4], Yet Another Great Ontology (YAGO; Max Planck Institute for Computer Science) [5], and FreeBase (Metaweb Technologies, Inc.) [6]. In China, there are Zhi Cube (Sogou), Zhi Xin (Baidu), zhishi.me (Shanghai Jiao Tong University) [7], and the GDM Lab Chinese KG project (Fudan University) [8]. In the medical field, the KG of medicine NKIMed [9] was developed by the Institute of Computer Technology of the Chinese Academy of Sciences, and the KG of Chinese medicine [10] was constructed by the Institute of Chinese Medicine Information of the Chinese Academy of Traditional Chinese Medicine. The Traditional Chinese Medicine Language System (TCMLS) is a relatively large semantic network for the KG of Chinese medicine [11], containing more than 100,000 concepts and 1 million semantic relations, which basically covers the conceptual system of TCM disciplines. The TCMLS was in the leading position of the TCM community in terms of its scale and completeness. Rotmensch et al [12] extracted positive mentions of diseases and symptoms (concepts) from structured and unstructured data in electronic medical records (EMRs) and used them to construct a health KG automatically.

#### Knowledge Graph Representation Learning

Graph neural networks (GNNs) are deep learning architectures for graph-structured data, which combine end-to-end learning with inductive reasoning. GNNs are promising research topics of AI, and they are expected to solve the problems of causal inference and interpretability that cannot be handled by traditional deep learning models. KG representation learning is a critical branch of the research on GNNs and plays a nontrivial role in knowledge acquisition and downstream application. KG representation learning consists of elements such as representation spaces (pointwise space, complex vector space, gaussian distribution, manifold, and group), scoring functions (distance-based and semantic-matching scoring functions), and encoding models (linear/bilinear, factorization models and neural networks).

Translational models leverage translational distances (eg, L1 or L2 norm) to model relations between head and tail entities. TransE is one of the representative translational models [13]. Dealing with 1-to-N, N-to-1, and N-to-N relations, TransE suffered from inefficiency problems in representing head or tail entities. To alleviate such problems, KGE models, including TransH [14], TransR [15], and TransD [16], were designed to impose translational distance constraints through different entity projection strategies. RotatE considers the embedding vectors of relations as rotations from source entities to target entities in a complex space [17].

The basic idea of factorization models is to decompose the matrix of each slice in a 3-way tensor into a product of entity vectors and relation matrices in the lower-dimensional space. The RESCAL model leveraged a relation-associated matrix to capture interactions between head and tail entities, which required a large number of parameters to model relations [18]. Therefore, vector forms of relations were introduced in DistMult [19] to decrease model parameters by restricting the interaction matrices to diagonal matrices. To increase the interactions between head and tail entities, a circular correlation operation was leveraged as the score function in the expressive HolE model [20]. Inspired by DistMult, the ComplEx model extended the representations of entities and relations by utilizing embedding vectors in a complex space [21]. An expressive KGE model named SimplE used 2 vectors for each entity to learn independent parameters through simplifying ComplEx by removing redundant computation [22].

In recent years, inspired by convolution operations, convolution-based KGE models, such as ConvE [23], ConvKB [24], and CapsE [25], were designed as different strategies to capture features between entities and relations for KG representation learning. A KGE model named knowledge base attention (KBAT) extended the graph attention (GAT) network by exploring the multihop representation of a given entity for representation aggregation via multihead attention and graph attention mechanisms [26]. The natural language pretraining model BERT [27] learned to integrate contextual information in the KG based on the representation of the transformer [28]. CoKE [29] used a transformer to encode edge and path sequences. These promising methods have attracted much attention due to the high efficiency of convolution in representation learning. CoKE aimed to learn the dynamic adaptive representations of entities and relations based on a rich graph structure context. Compared with static representations, the performance of contextual models is state of the art, since the representations combined with contextual semantic information are richer and more flexible. Despite the use of a transformer, CoKE was still parameter-efficient to obtain competitive performance with fewer parameters. The comparison of the KG representation learning models is shown in Table 1.

**Table 1:** Comparison of baseline KGE^a^ models.

Model			Scoring function f_r_(h,t)		Entity and relation embedding
**Translational model**					
	TransE [13]	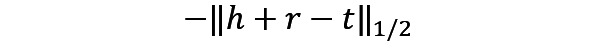		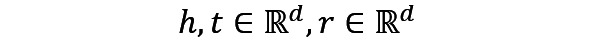	
	TransH [14]	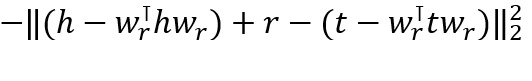		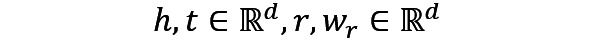	
	TransR [15]	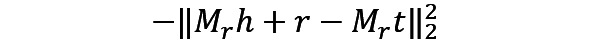		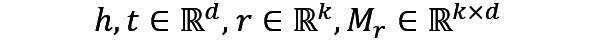	
	TransD [16]	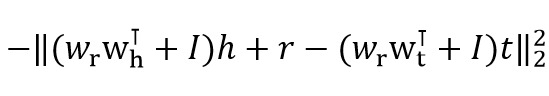		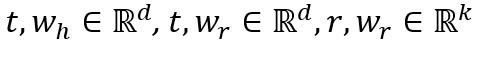	
**Linear/bilinear model**					
	SimplE [22]	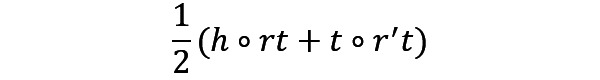		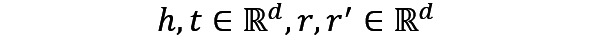	
	HolE [20]	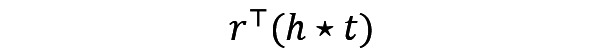		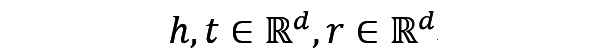	
**Rotational model**					
	QuatE [30]	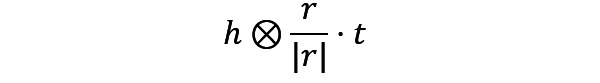		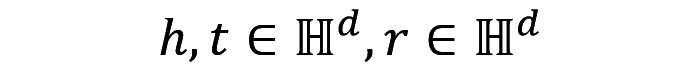	
	RotatE [17]	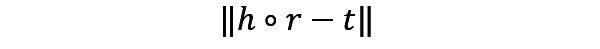		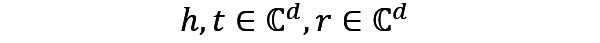	
**Convolutional neural network**					
	ConvE [23]	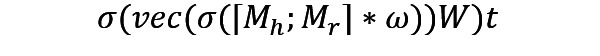		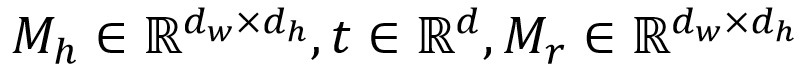	
	ConvKB [24]	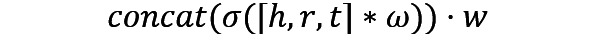		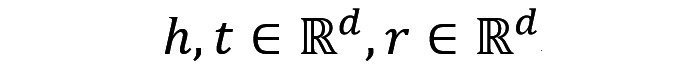	
**GNN^b^**					
	KBAT^c^ [26]	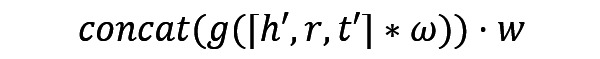		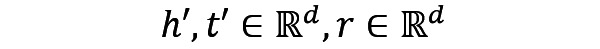	
**Neural network transformer**					
	CoKE^d^ [29]	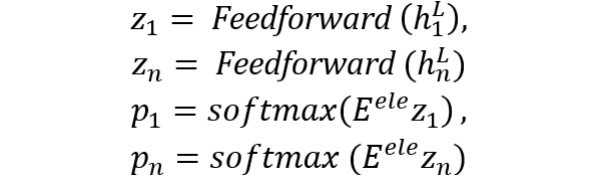		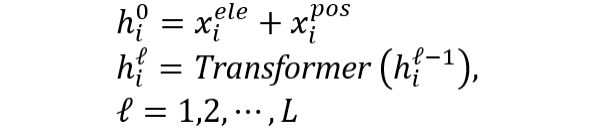	

^a^KGE: knowledge graph embedding.

^b^GNN: graph neural network.

^c^KBAT: knowledge base attention.

^d^CoKE: Contextualized Knowledge Graph Embedding.

#### Application of Medical Knowledge Graphs

The hot topics related to the application of medical KGs are KG-fused multimodal information diagnosis, KGE-based text classification, and knowledge inference–based medical question answering and assisted diagnosis. Shen et al [31] reused the existing knowledge base to build a high-quality KG and designed a prediction model to explore pharmacology and KG features. The model allowed the user to gain a better understanding of the drug properties from a drug similarity perspective and insights that were not easily observed in individual drugs. Zheng et al [32] took advantage of 4 kinds of modality data (X-ray images, computed tomography [CT] images, ultrasound images, and text descriptions of diagnoses) to construct a KG. The model leveraged multimodal KG attention embedding for diagnosis of COVID-19. The experimental results demonstrated that it was essential to capture and join the importance of single- and multilevel modality information in a multimodal KG. Li et al [33] designed an AI-powered voice assistant by constructing a comprehensive knowledge base with ontologies of defined Alzheimer disease and related dementia (ADRD) diet care and user profiles. They extended the model with external KGs, such as FoodData Central and DrugBank, which personalized ADRD diet services provided through a semantics-based KG search and reasoning engine.

With the development of deep learning methods, diagnostic decisions have become interpretable. Theoretically, rule-based engines may infinitely approximate the performance of nonlinear classifiers by mining the expanded knowledge. In other words, through the integration of interpretable knowledge rules, rule-based engines may approximate the performance of deep learning models. Through deep mining of rules, the clinical assisted decision-making system may be able to perform multiple rounds of rule expansion under dynamic thresholds and further extend the capability of decision-making based on existing knowledge.

## Methods

### TCM Knowledge Graphs

To construct a TCM KG (Table 2) for ordinary usage, such as disease diagnosis and treatment assistance, we cleaned the EMR data set of diagnosis and treatment of TCM diseases and represented the relations of entities in triples. For instance, given a description text of insulin resistance as a mechanism of type 2 diabetes, the entities and relations in the sentence were extracted and organized into a disease mechanism triple of (insulin resistance, mechanism=>disease, diabetes). A KG was defined as G=(E,R,S), where entities, relations, and triples are 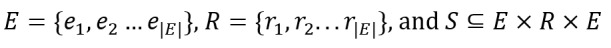, respectively, and |E| and |R| are the counts of entities and relations. The triples consisted of entities, relations, describing concepts, or attributes.

Traditional KGE models are designed to learn static representations of entities and relations. The features of graph contexts are obtained by representing neighbor entities and relations. Different meanings are expressed by entities and relations in diverse contexts, as words appear in different textual contexts. Multihop relations (ie, paths between entities) can provide rich contextual features for reasoning in KG [29]. Existing work [34] shows that multihop relation paths contain rich inference patterns between entities. Since not all relation paths are reliable, we designed a causal-constraint algorithm to filter the reliability of relation paths. Relation paths were represented via semantic composition of relation embeddings. The screened multihop relations were extended to triple alternative combinations.

**Figure 1:**
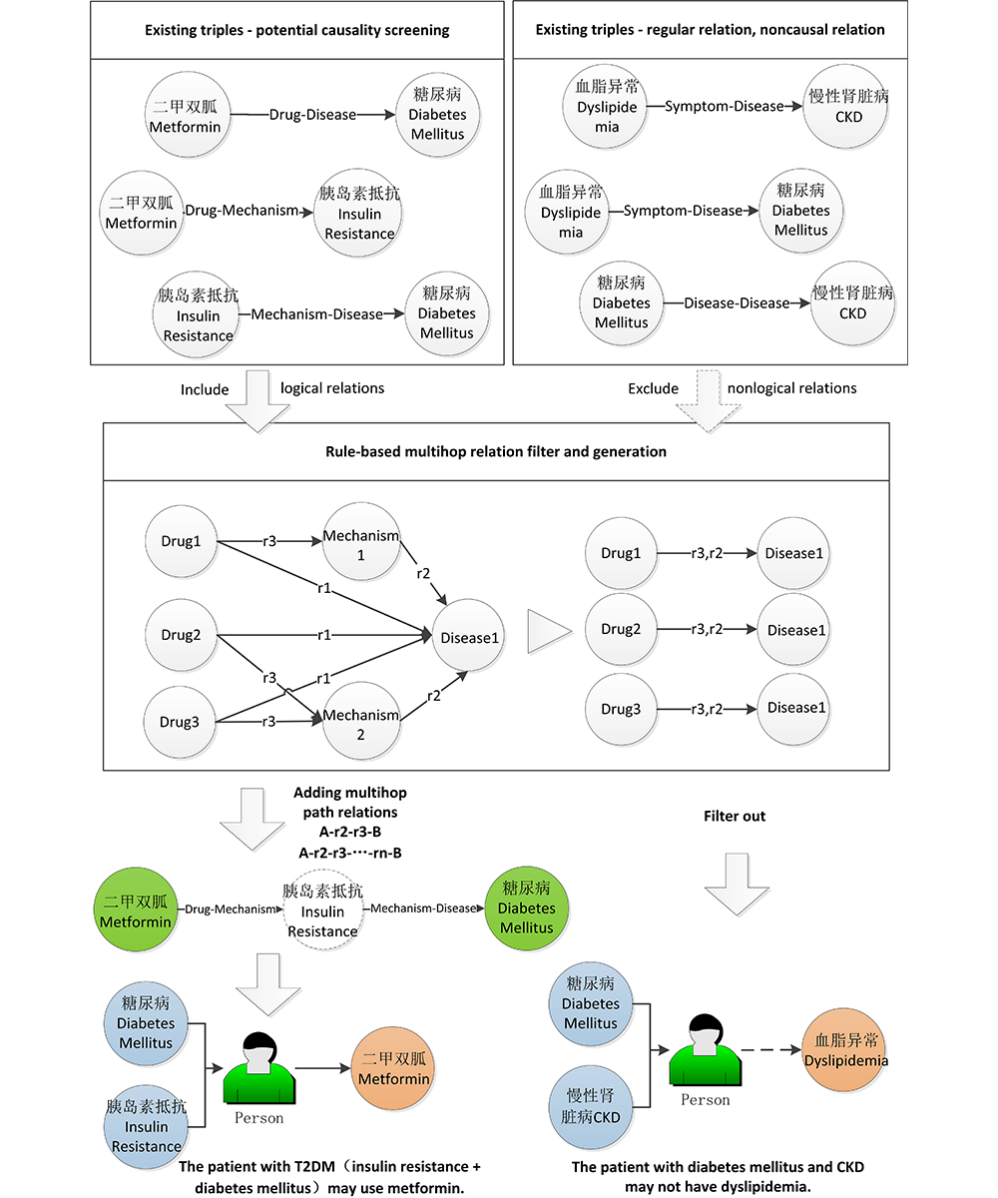
Positive and negative examples of multihop relation filtering and generation. CKD: chronic kidney disease; T2DM: type 2 diabetes mellitus.

The rules for screening potential multihop causal relations are shown in Figure 1. For example, there exist triples (*insulin resistance*, *treat*, *diabetes mellitus*) and (*metformin*, *mechanism*, *insulin resistance*) in a clinical KG describing the relations between clinical mechanism and disease (or drug) as a positive example in the figure. The relations can be inferred as the causal multihop relation between a drug and a disease by the rules drug=>mechanism and mechanism=>disease, indicating that metformin can treat insulin-resistant diabetes. The triples (*dyslipidemia*, *symptom*, *diabetes mellitus*) and (*dyslipidemia*, *symptom*, *CKD* [where CKD refers to chronic kidney disease]) co-occurred and thus could not reflect the causal relation between diabetes mellitus and CKD or dyslipidemia. Such negative triples were screened according to the rules.

An example of a casual multihop relation of TCM disease (abdominal mass)=>mechanism (*phlegm dampness*, *toxin*, *blood stasis*)–mechanism=>mechanism (*clearing heat-toxin*, *eliminating dampness*)–disease=>drug (*root of Chinese* Pulsatilla) can be inferred according to the rules (*abdominal mass*, *disease=>drug*, *phlegm dampness*, *toxin*, *blood stasis*), (*phlegm dampness*, *toxin*, *blood stasis*, *mechanism=>mechanism*, *clearing heat-toxin*, *eliminating dampness*), and (*abdominal mass*, *disease=>drug*, *root of Chinese* Pulsatilla). In other words, casual multihop relations of TCM can be inferred, which conform to the cognition of diseases–syndrome–principle–method–recipe–medicines of TCM, including the aforementioned path disease=>mechanism=>treatment=>drug.

The semantics of the entities *diabetes mellitus* and *metformin* were enriched by the embeddings of the 2-hop path inferred by triples (*metformin*, *mechanism*, *insulin resistance*) and (*insulin resistance*, *treat*, *diabetes mellitus*). To represent multihop relations, given the 2-hop path from the entity *metformin* to *diabetes* *mellitus*, triple forms (*metformin*, *mechanism-treat*, *diabetes mellitus*) were used for consistency. Since the multihop features were integrated, the representations of entities and relations tended to have strong inference capability, which facilitated entity link prediction. The KG was represented as textual triples that described multihop relations of entities.

**Table 2:** Overview of the TCM^a^ KG^b^.

Relation name	Heads, n	Tails, n	Triples, n
symptom=>symptom	8101	8544	51,345
disease=>symptom	12,225	15,071	133,648
disease=>drug	12,650	11,526	84,524
mechanism=>mechanism	527	51	590
symptom=>drug	3941	6145	24,724
symptom=>mechanism	6544	1096	10,906
symptom=>disease	8101	10,391	87,651
mechanism=>department	1908	65	4408
symptom=>body parts	318	85	548
mechanism=>body parts	2217	72	3221
mechanism=>symptom	2147	4191	16,377
symptom=>department	10,157	178	24,870
disease=>mechanism	7774	5304	46,425
disease=>body parts	7607	110	13,505
disease=>department	14,484	284	40,762
disease=>disease	9728	10,545	40,575
mechanism =>disease	2228	5443	20,621

^a^TCM: traditional Chinese medicine.

^b^KG: knowledge graph.

### Knowledge Graph Representation Framework

**Figure 2:**
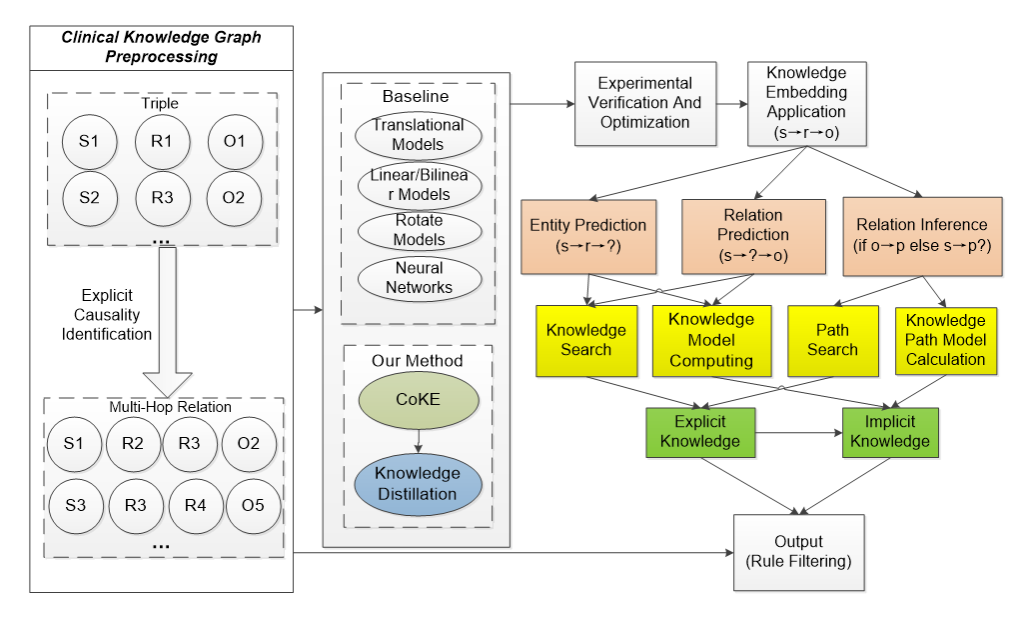
Proposed framework of TCM KG representation learning. CoKE: Contextualized Knowledge Graph Embedding; KG: knowledge graph; TCM: traditional Chinese medicine.

After preprocessing of the TCM KGs, we applied a CoKE-based KG representation learning model based on a diagnosis and treatment KG of Chinese and Western medicine and proposed a new KG representation framework. Compared with popular knowledge representation learning models, such as TransE and KBAT, our framework features the fusion of CoKE and multihop relations. The framework was verified with downstream applications, such as assisted decision-making and question answering, as shown in Figure 2.

### Entity Link Prediction

The CoKE model was leveraged as the base model in this paper. The BERT model was leveraged to learn contextualized embeddings of entities and relations in CoKE. The input sequence X = (x_1_, x_2_, …, x_n_) consisted of the embeddings of a head entity x_1_ and a tail entity x_n_, while the embeddings of relations were denoted as x_2_ from x_n–1_. Given x_i_from the input sequence, the hidden representation h_i_ was expressed as Equation 1:



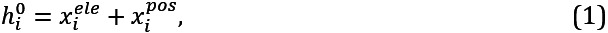



where 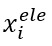 is the embedding of an element and 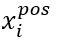 is the positional embedding of an element. The former was used to identify the current entities or relations in 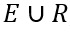, and the latter presented the positional features of the element in the sequence. The constructed hidden representations were fed into transformer encoders of L layers as Equation 2:



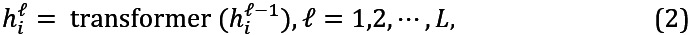



where 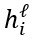 is the hidden representation of x_i_ at the l-th layer of the encoder. A multihead self-attention mechanism was leveraged by the transformer, which allowed each element to attend to other elements in the sequence effectively for contextual feature modeling. As the use of transformers has become ubiquitous recently, we omitted a detailed description of the transformer. The final hidden representations 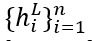 are representations for entities and relations within the sequence X. The learned representations were naturally contextualized and automatically adaptive to the input.

### Multihop Relational Representation Learning

Given a triple (s,r,o) in a KG, the contexts between a head and a tail entity can be described as an edge and a path. An edge s→r→o is presented as a sequence that can be viewed as a triple. For instance, an edge *metformin*→*mechanism*→*insulin resistance* can form a triple (*metformin*, *mechanism*, *insulin resistance*) equivalently. As the basic unit of a KG, an edge (or a triple) is the simplest form of a graph context describing an entity. Another context is a path s→r_1_→…→r_k_→o as a sequence consisted of head and tail entities and a list of linked relations between them. For instance, the path 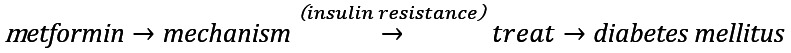 describes multihop relations between the head entity *metformin* and the tail entity *diabetes* *mellitus*, where *insulin resistance* is the intermediate entity in the path, while *mechanism* and *treat* are the relations. The path can be expressed as a triple (*metformin*, *mechanism-treat*, *diabetes mellitus*). Consisting of contextual features of entities, the multihop path representation can be leveraged for reasoning in a KG.

To verify the effectiveness of the model, experiments of entity link prediction in knowledge graph completion (KGC) [35] and multihop relation representation learning were conducted. Entity link prediction refers to a task that predicts missing target entities of triples (h, r, ?) and (?, r, t) with a candidate entity set by semantic constraints of KGE models. PathQuery answering [36] was utilized in the experiments of multihop relation representation learning. Given a source entity s and a relation path p, a set of target entities that were inferred from the source entity s via the path p was predicted.

In entity link prediction, our model was trained to predict missing target entities, given a context in the KG, answering 1-hop or multihop queries. Different strategies were considered to train our model with respect to the cases of edges and paths. Each edge s→r→o is associated with 2 instances ?→r→o and s→r→?, which can be regarded as 1-hop query answering. For instance, *metformin*→*mechanism*→*?* is to answer the query, What is the mechanism of m*etformin*? Similarly, each path s→r_1_→…→r_k_→o is also associated with 2 instances, one to predict s and the other to predict o, which can be viewed as multihop question answering. For instance, 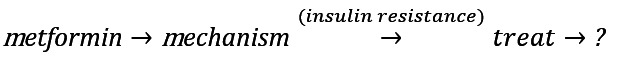 is to answer the query, What disease can be treated by the mechanism of *metformin*?

In the training procedure, edges or paths were unified as an input sequence X = (x_1_, x_2_, …, x_n_). Two instances were created by replacing x_1_ with a special token [MASK] for s prediction and by replacing x_n_ with [MASK] for o prediction. The masked sequence was fed into the transformer encoding blocks to obtain the final hidden representation for target entity prediction.

As in the BERT model, the representations of the masked entities were fed into a feedforward neural network and a standard Softmax layer was leveraged for classification (Equation 3):



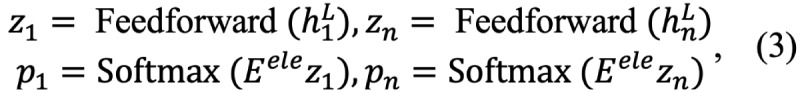



where z_1_ and z_n_ are the representations of h^L^_1_ and h^L^_n_ produced by the feedforward layer, while 
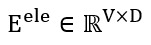
 is a matrix shared with the input element embedding matrix for classification. D is the hidden size, V is the size of the entity vocabulary, and p_1_ and p_n_ are the predicted distributions of target entities s and o. Cross-entropy loss was leveraged as the loss function for classification (Equation 4):



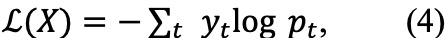



where y_t_ and p_t_ are the t-th components of the 1-hot label vector y and the distribution vector p, respectively. A label-smoothing strategy was leveraged to lessen the restriction of 1-hot labels. In other words, the value of the target entity was set to ε, while y_t_ = (1 – ε)/(V – 1) for incorrect entities in the candidate entity set.

### Knowledge Distillation

**Figure 3:**
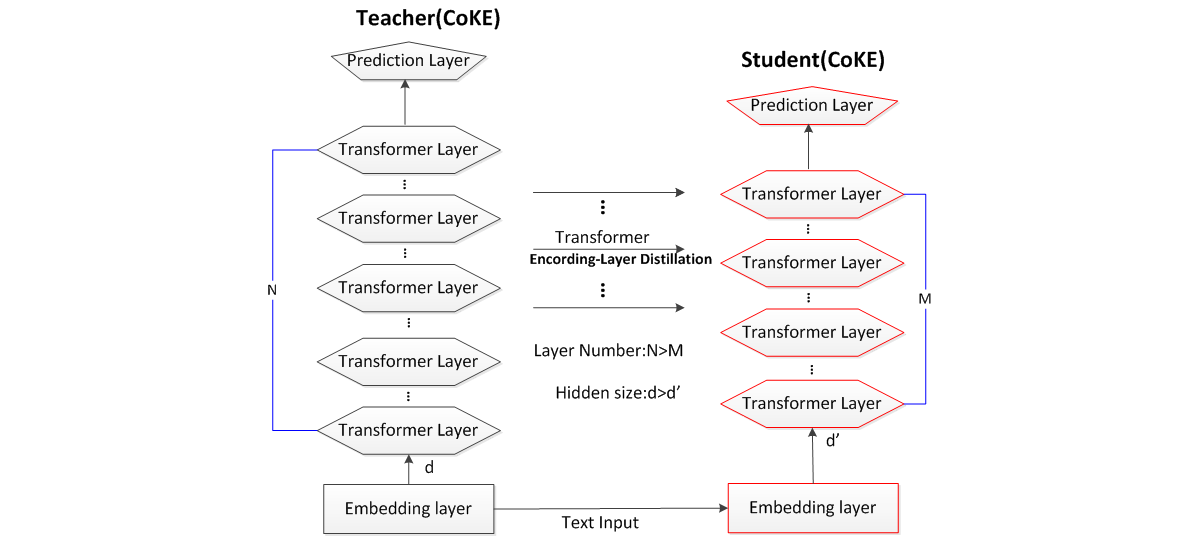
Architecture of CoKE-distillation. CoKE: Contextualized Knowledge Graph Embedding.

Inspired by the idea of TinyBERT [37] for knowledge distillation, our model CoKE-distillation contains a teacher and a student model for knowledge distillation, as shown in Figure 3.

Our proposed CoKE-distillation model consists of 3 levels of distillation: embedding layer distillation, transformer -layer distillation, and prediction layer distillation. At the embedding layer distillation level, the embedding matrices of the student and teacher model are constrained by the mean-square error (MSE) loss (Equation 5):



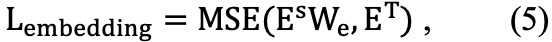



where 
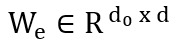
 is a trainable linear transformation matrix to project the embedding of the student model into the semantic space of the teacher model. The embedding matrices of the student and teacher models are denoted by 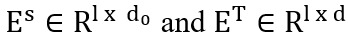, where l is length of the sequence, d_0_ is the size of the embeddings of the teacher model, and d is the size of the embeddings of the student model.

At the level of transformer layer distillation, the CoKE-distillation model distills knowledge in k-layer intervals. For instance, if the student model has 4 layers, a transformer loss is calculated every 3 layers, since the teacher model has 12 layers. The first layer of the student model corresponds to the third layer of the teacher model, while the second layer of the student model corresponds to the sixth layer of the teacher model and so on. The transformer loss of each layer is divided into 2 parts, attention-based knowledge distillation and implicit state–based knowledge distillation. The loss of each layer consists of an attention-based knowledge distillation loss and a hidden state-based knowledge distillation loss.

The attention-based knowledge distillation loss is expressed as Equation 6:



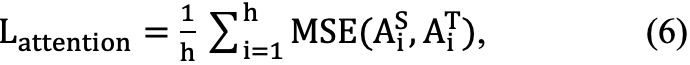



where h is the number of attention heads, 
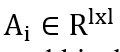
 refers to the attention matrix corresponding to the i-th head of the teacher or the student, and l is the length of the input text.

The hidden state-based knowledge distillation loss is expressed as Equation 7:



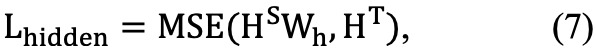



where the matrices 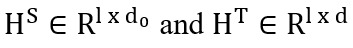 refer to the hidden representations of student and teacher models, respectively. At the level of prediction layer distillation, prediction loss is shown as Equation 8:



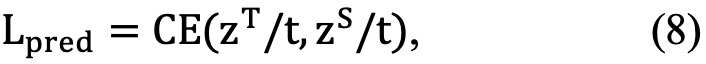



where z^T^ and z^S^ are the logit vectors predicted by the student and the teacher respectively, CE means the cross-entropy loss, and t means the temperature value. In our experiment, t was set to .

## Results

### Data Set

To evaluate the proposed model, a widely used standard data set FB15k-237 [38] was used, which is a subset of the Freebase knowledge base [6] with 14,541 entities and 237 relations. Due to redundant relations existing in the FB15k data set, FB15K-237 removes the inverse relations, preventing models from directly inferring target entities by inverse relations. The FB15k-237 data set is randomly divided into 3 sets (training, validation, and test sets), with 272,115 triples in the training set, 17,535 triples in the validation set, and 20,466 triples in the test set.

We constructed a medical diagnosis and treatment data set of TCM, called TCMdt, consisting of entities and relations as triples. The data set contained 17 kinds of relations, 59,882 entities, and 604,700 triples without repetitive and inverse relations. There were 3811 kinds of N–1 relations, such as relation combinations *mechanism-body parts* and *mechanism-mechanism*. The rest of the relations were N–N relations, 600,868 in total. There were no 1–1 and 1–N relations in the data set. The data set was divided into a training, a validation and a test set, containing 59,882 entities and 17 relations in total. The details of the FB15k-237 and TCMdt data sets are shown as Table 3.

The hypertension data set (Table 4) in TCM for the multilabel modeling task was used in our experiment to evaluate the effectiveness of KGE learning. TCM has been used for the diagnosis of hypertension and has significant advantages. Symptom analysis and modeling of TCM provide a way for clinicians to accurately and efficiently diagnose hypertension. In this study, the initial data were collected from trained practitioners and clinical practitioners. Details of 928 cases of hypertension were collected from the clinical departments of the Guangdong Provincial Hospital, with both inpatient and outpatient medical records from the Liwan district [39]. All cases with incomplete information were removed from the data set, and the remaining 886 (95.47%) cases were used for analysis in this study.

Each case in the data set had 129 dimensions of TCM symptom features and syndrome diagnosis labels in 1-hot format. Each case had 2-5 labels of TCM syndrome diagnosis reidentified by trained clinicians. The KGE of the syndrome entities and the symptom vectors and matrix were constructed from the aforementioned TCMdt data set.

**Table 3:** Statistics of the FB15k-237 data set and the constructed TCMdt data set.

Data set	Entities, n	Relations, n	Triples in the training set, n	Triples in the validation set, n	Triples in the test set, n
FB15k-237	14,541	237	272,115	17,535	20,446
TCMdt	59,882	17	544,230‬	30,235	30,235

**Table 4:** Statistics of the hypertension data set in TCM^a^.

Features, n	Classes, n	Total cases, N	Validation
121	8	886	10-fold cross-validation

^a^TCM: traditional Chinese medicine.

### Baselines

Baseline methods were used for comparison in the experiments, including translational models, bilinear models, a rotational model, a GNN, and a transformer-based model. The details of the models and their types are shown in Table 5.

**Table 5:** Baseline methods for KG^a^ representation learning.

Type of model	Models
Translational model	TransE [13], TranH [14], TransR [15], TransD [16]
Linear/bilinear model	ComplEx [21], DistMult [19], SimplE [22]
Rotational model	RotatE [17]
GNN^b^	KBAT^c^ [26]
Transformer-based model	CoKE^d^ [29]

^a^KGE: knowledge graph.

^b^GNN: graph neural network.

^c^KBAT: knowledge base attention.

^d^CoKE: Contextualized Knowledge Graph Embedding.

### Evaluation Metrics

With respect to the evaluation metrics, Sun et al [40] found that some high performance can be attributed to the inappropriate evaluation protocols and proposed an evaluation protocol to address this problem. The proposed protocol was more robust to handle bias in the model, which could substantially affect the final results. Ruffinelli et al [41] conducted systematic experiments on the training methods used in various KGE models and found that some early models (eg, RESCAL) can outperform the state-of-the-art models, after adjusting the training strategies and exploring a larger search space of hyperparameters. This indicated that the performance improvement of the models might not reflect their advantage, since the training strategies might play a critical role. Therefore, we established a unified evaluation standard to mine the valuable ideas and superiority of the models.

We used the mean reciprocal rank (MRR) and Hits@N, which are frequently used evaluation metrics for link prediction task in KGs (Equations 9 and 10). Applying the filtered settings given by Wang et al [14], the rank of the head or tail entities in a test triple (e_i_, r_k_, e_j_) was computed within a filtered entity set. The filtered entity set contained entities that could be used to generate valid triples without valid head or tail entities in the training set. A large value of the MRR indicates that the KGE model have the capability of precise entity representation, while Hits@N denotes a rate of head and tail entities that rank within N (1, 3, or 10) empirically.



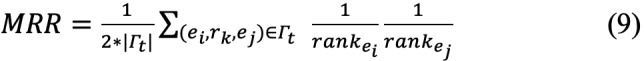





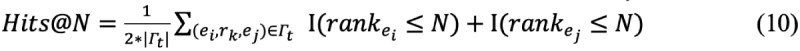



In the equations, |Γ_t_| is the size of testing triple set Γ_t_ and I(·) is an indicator function, while 
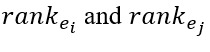
 denote values of ranks for a head and a tail entity e_i_ and e_j_, respectively.

### Model Performances

During the comparison, we evaluated the models with embedding vectors of 256, 512, 1024, and 2048 dimensions and sufficient iterations to ensure the obtained embeddings were qualified for the sake of the downstream task. The results are shown in Tables 6 and 7. Compared with the baseline models, the CoKE model showed a competitive performance on both the standard data set and the constructed TCMdt data set. The CoKE model had the highest MRR and CoKE-multihop model had the best Hits@10. The CoKE-multihop-distillation model still showed a competitive performance on the MRR and HIT@10 compared to the CoKE model.

To evaluate the effectiveness of the KGE learning, 10-fold cross-validation was used in the multilabel modeling task experiments. Compared with typical models multilabel k nearest neighbors (MLKNN), RandomForest-RAkEL (where RAkEL refers to random k-labelsets), LogisticRegression-RAkEL, and deep neural network (DNN) [42], the proposed model outperformed the baseline models on metrics precision, recall, and the F1 score, as shown in Table 8. In addition, multilabel models with KGE had better performance than those without KGE. The results demonstrate that learned KGE is capable of improving the performance of deep learning models.

**Figure 4:**
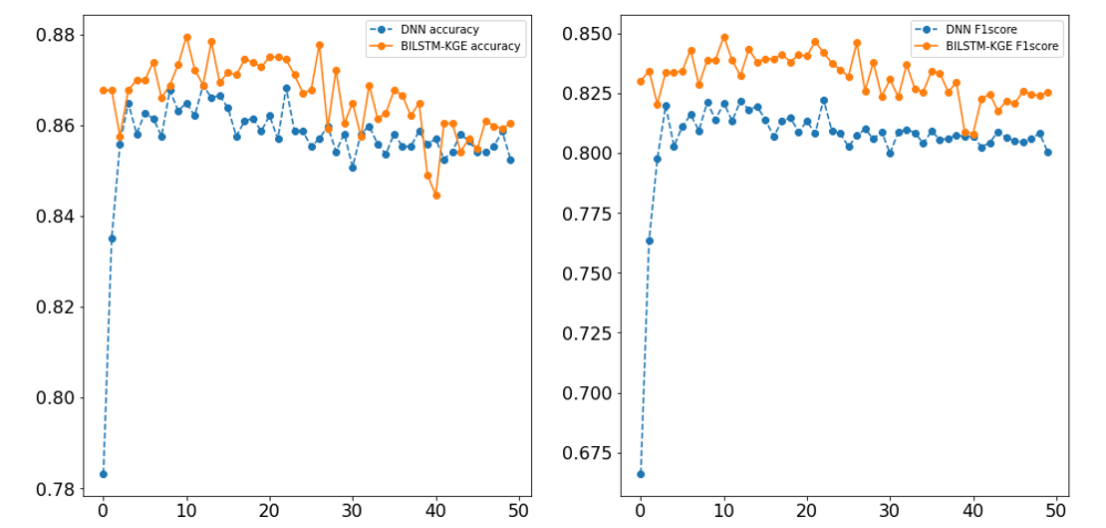
Performances of DNN and DNN+BILSTM-KGE. BILSTM: bidirectional long short-term memory; DNN: deep neural network; KGE: knowledge graph embedding.

As shown in Figure 4, the DNN+BILSTM-KGE (where BILSTM refers to bidirectional long short-term memory) outperformed the DNN on evaluation metrics (eg, precision and F1 score) in the training procedure. Compared with the DNN, the average precision and F1 score of DNN+BILSTM-KGE showed improvement, with the Hamming loss significantly decreasing for the first 50 iterations.

**Table 6:** Performance comparison of link prediction on the FB15k-237 data set.

Models	MRR^a^	Hits@N		
		@10	@3	@1
TransE	0.296	0.499	0.330	0.196
SimplE	0.306	0.496	0.341	0.212
RotatE	0.314	0.505	0.347	0.221
ComplEx	0.296	0.489	0.333	0.200
DistMult	0.309	0.506	0.346	0.211
KBAT^b^	0.103	0.337	0.248	0.103
ConvKB	0.407	0.527	0.333	0.200
CoKE^c^	0.362	0.550	0.400	0.269

^a^MSE: mean-square error.

^b^KBAT: knowledge base attention.

^c^CoKE: Contextualized Knowledge Graph Embedding.

**Table 7:** Performance comparison of link prediction on the TCMdt data set.

Models	MRR^a^	Hits@N		
		@10	@3	@1
TransE	0.243	0.428	0.279	0.150
SimplE	0.162	0.436	0.222	0.113
RotatE	0.146	0.424	0.193	0.090
ComplEx	0.137	0.411	0.177	0.080
DistMult	0.164	0.438	0.223	0.117
ConvKB	0.271	0.464	0.302	0.192
CoKE^b^	0.332	0.491	0.365	0.250
KBAT^c^	0.129	0.369	0.178	0.088
CoKE-multihop	0.251	0.515	0.278	0.261
CoKE-multihop-distillation	0.32	0.483	0.374	0.260

^a^MSE: mean-square error.

^b^KBAT: knowledge base attention.

^c^CoKE: Contextualized Knowledge Graph Embedding.

**Table 8:** Results of 10-fold cross-validation of deep learning multilabel models.

Index		Precision	Recall	F1 score
**MLKNN^a^ (Hamming loss=0.186; best parameter: K=26)**				
	Micro-avg	0.810	0.710	0.760
	Macro-avg	0.800	0.610	0.660
**RandomForest-RAkEL^b^ (Hamming loss=0.186; best parameter: n_estimators=800)**				
	Micro-avg	0.790	0.740	0.760
	Macro-avg	0.760	0.640	0.670
**LogisticRegression-RAkEL (Hamming loss=0.173; best parameter: C=0.5)**				
	Micro-avg	0.810	0.750	0.780
	Macro-avg	0.760	0.660	0.700
**DNN^c^ (Hamming loss=0.186; best parameters: hidden=500, layer=3)**				
	Micro-avg	0.790	0.740	0.760
	Macro-avg	0.750	0.670	0.700
**DNN+LSTM^d^-KGE^e^ (Hamming loss=0.167; best parameters: hidden=500, layer=3, LSTM=128)**				
	Micro-avg	0.800	0.790	0.790
	Macro-avg	0.740	0.740	0.740
**DNN+BILSTM^f^-KGE (Hamming loss=0.127; best parameter: LSTM=128)**				
	Micro-avg	0.860	0.820	0.840
	Macro-avg	0.810	0.770	0.790

^a^MLKNN: multilabel k nearest neighbors.

^b^RAkEL: random k-labelsets.

^c^DNN: deep neural network.

^d^LSTM: long short-term memory.

^e^KGE: knowledge graph embedding.

^f^BILSTM: bidirectional long short-term memory.

**Figure 5:**
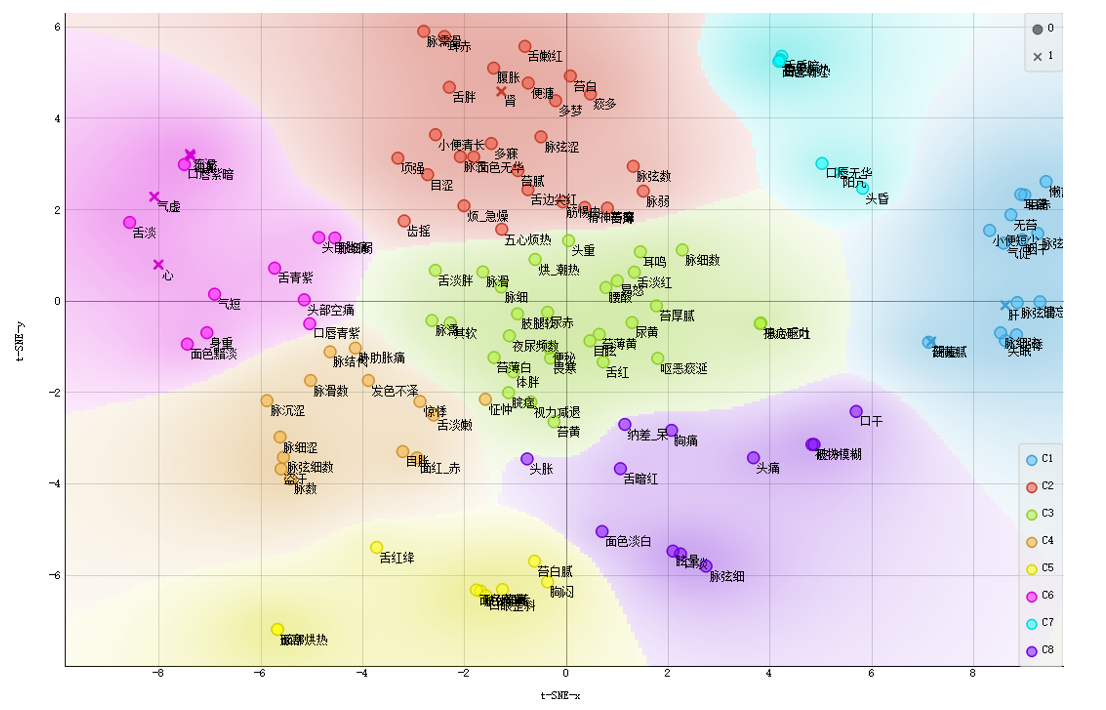
Learned representations of entity visualization.

Learned representations of entities were visualized by t-SNE, as shown in Figure 5. Symptoms and TCM syndrome elements are denoted by ○ and X, respectively. The representation distribution conformed to theoretical common sense in TCM with obvious boundaries (ie, silhouette score>0.44) between different classes of TCM syndromes. Intuitively, the learned representations preserved the semantic information about TCM syndromes by using the proposed KGE learning methods. In addition, the relation between entities *Yang hyperactivity* and *dizziness* was similar to the relation between entities *liver depression* and *stringy pulse*, indicating that the semantic constraint of translational distance is preserved after training. The results show that representations learned by the proposed KGE learning method are capable of providing semantic information in TCM.

## Discussion

### Principal Findings

The experiments show that the CoKE model has a more stable performance and can be used for improving downstream tasks. We assume that downstream tasks may be improved by KGE learning, since semantic information provided by KGE is preserved in learned representations of missing entities and relations in a KGC task. KGE is suitable to be applied in scenarios that suffer from incompleteness issues, including knowledge discovery for diagnosis and treatment and assisted decision-making in TCM. Based on the clinical KGE model, we automatically extracted the information about dominant diseases treated by Chinese physicians, evidence, symptoms, theories, treatment methods, prescriptions, medicines, and concept mappings according to the definition of clinical knowledge ontology by the physicians. Inspired by Luo et al [43] and Jin et al [44], the triples in a clinical KG are used to learn a personalized KGE model of Chinese physicians.

The problem of incompleteness of a KG is alleviated by entity link prediction of the personalized KGE model. Through the visualization of the KG, our system assists experts in identifying and expanding the potential relations and neighbors of entities in order to obtain explicitness of the implicit knowledge. Through multiple iterations of embedded learning, the KGE model is suitable for treatment decision-making of Chinese physicians. The theories, treatment methods, prescriptions, capability of cause-effect reasoning, and interpretability are enhanced.

**Figure 6:**
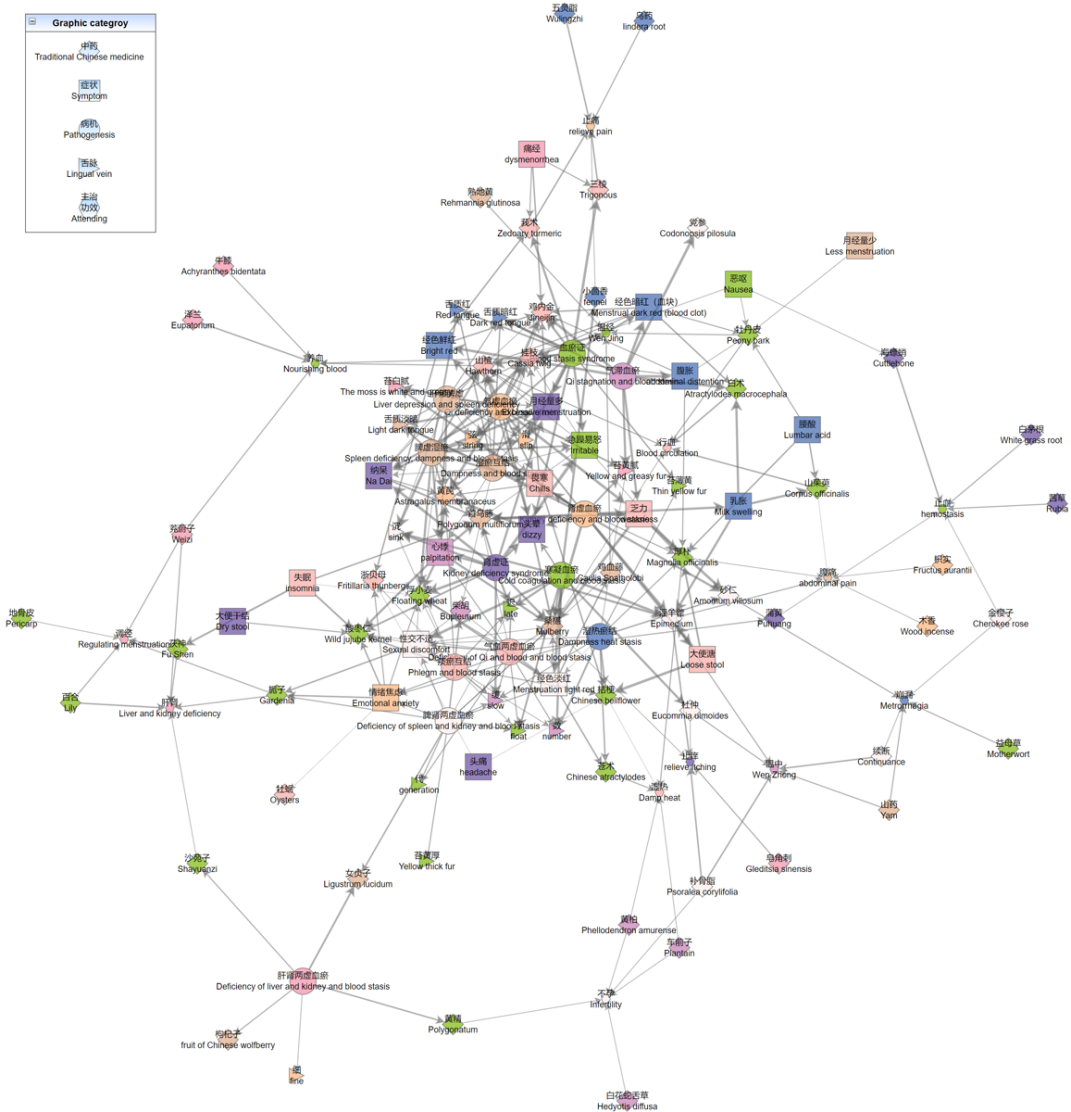
Visualization of a personalized KG that consists of theories, treatment methods, prescriptions, and medicines of EM in TCM. EM: endometriosis; KG: knowledge graph; TCM: traditional Chinese medicine.

Consisting of theories, treatment methods, prescriptions, and medicines of endometriosis (EM) in TCM, the visualization of our KG is shown in Figure 6. A personalized KG for gynecology is constructed to assist experts in knowledge discovery and decision-making. The thickness of the arrows represents the strength of the potential causality, and the size of the nodes represents their importance in the KG of EM in gynecology. Our system clusters the nodes and represents them with different colors of the clusters. Different shapes of nodes represent different entity types.

We referred to a large amount of ancient and modern literature and the diagnosis and treatment data of Chinese and Western medicine, combined with the techniques of entity extraction and causality extraction in natural language processing. According to the definition of domain knowledge by Chinese physicians, valid entities and relations from real cases include the names of TCM diseases, Chinese medicines and prescriptions, tests and examinations, names of Western medicines and diseases, TCM symptoms, and hospital departments. In the training procedure, the weights of the CoKE model were updated until convergence in order to generate embedding vectors that captured semantic features for clinical interpretability. The proposed model can be applied for personalized recommendations of Chinese physicians, question answering, and optimization of diagnostic models.

**Figure 7:**
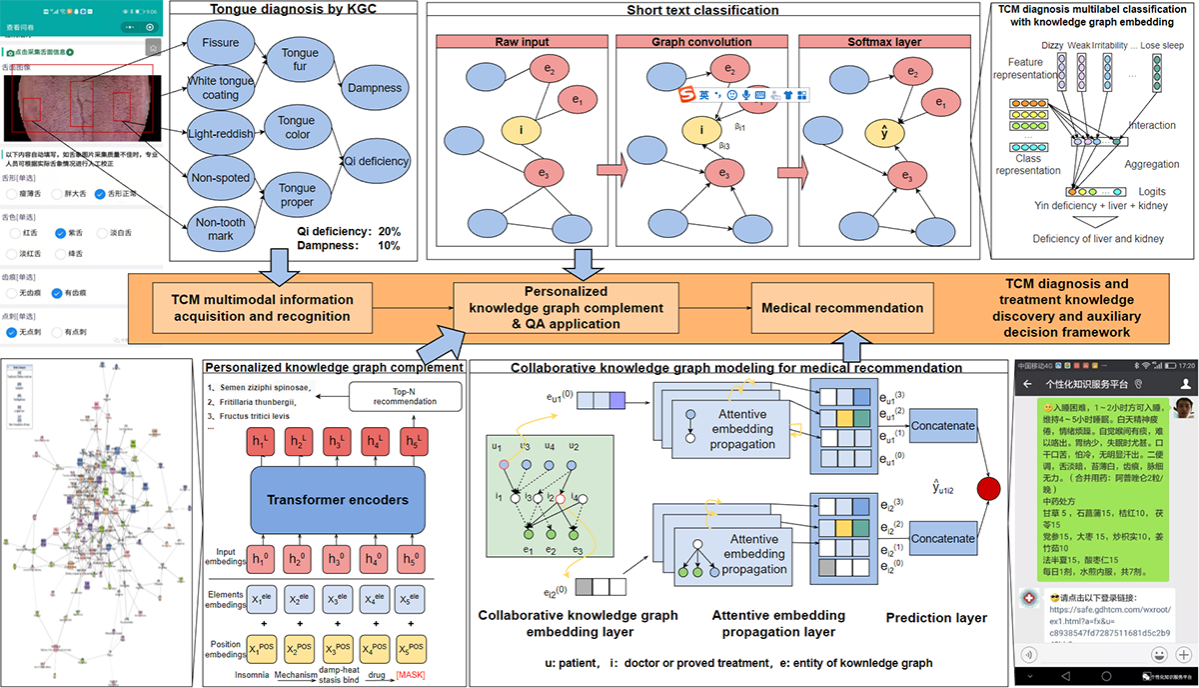
Application of the framework to knowledge discovery and decision-making in TCM. CKG: collaborative knowledge graph; TCM: traditional Chinese medicine; QA: question and answer.

Inspired by the heterogeneous network representation learning model [45], a framework for knowledge discovery and decision-making in TCM was proposed, as shown in Figure 7.

For medical recommendation and assisted decision-making, the first step is to collect objective information about the four diagnostic methods. The clinical KG incorporates multimodal information recognized from tongue and facial diagnosis equipment, which can be used to improve the performance of models, even in few-shot learning scenarios. KGs can be used to effectively solve the problems of sparsity and cold start in recommendation systems. Integrating KGs into recommendation systems as external information facilitates the systems with common-sense reasoning capability. Based on the powerful capability of information aggregation and the inference of GNNs, we designed a recommendation system to recommend symptoms, diseases, and Chinese physicians, which effectively improves the performance of recommendations. In addition, the information propagation and inference capability of GNNs also provide interpretability for the results of recommendations.

The model can be used for high-quality assisted decision-making in diagnosis and treatment based on multimodal information and specialty questionnaires. Our system helps practitioners and patients efficiently build online profiles, which enhances the research value of clinical cases. Constructed from natural language, KGs have a strong connection to text mining. KGE can be used to boost the performance of models for text classification and generation. For example, KGE can be leveraged for entity disambiguation when answering the question of what glucose-lowering drug is better for obese diabetics. Similar to link prediction, knowledge inference in question answering infers new relations between entities, given a KG, which is often a multihop relation inference process. For instance, the question can be viewed as a query 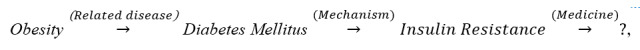 which can be predicted by PathQuery answering of CoKE for medicine recommendation to obtain related medicines, including *metformin* [46-49].

### Conclusion

In this paper, a KG-fused multihop relational adaptive CoKE framework was proposed for screening enhancement, knowledge complement, knowledge inference, and knowledge distillation. The superiority of the model in knowledge discovery and assisted decision-making in TCM was shown in experiments and clinical practice. TCM is a systematic discipline focusing on inheritance and practice. A large amount of knowledge is hidden in the ancient literature and experimental cases of Chinese physicians, which can be mined by researchers. In the future, we aim to improve the quality of the intelligent system of human-machine collaborative KGs in TCM. More in-depth research will be conducted on the knowledge fusion of heterogeneous GNNs, complex inference of KGs with GNNs, and interpretable learning of GNNs.

## Abbreviations

ADRD: Alzheimer disease and related dementia
AI: artificial intelligence
BILSTM: bidirectional long short-term memory
CKD: chronic kidney disease
CoKE: Contextualized Knowledge Graph Embedding
DNN: deep neural network
EM: endometriosis
EMR: electronic medical record
GNN: graph neural network
KBAT: knowledge base attention
KG: knowledge graph
KGC: knowledge graph completion
KGE: knowledge graph embedding
MLKNN: multilabel k nearest neighbors
MRR: mean reciprocal rank
MSE: mean-square error
RAkEL: random k-labelsets
TCM: traditional Chinese medicine
TCMLS: Traditional Chinese Medicine Language System
